# A Case Report of an Unusual Presentation of Plexiform Epithelioid Schwannoma

**DOI:** 10.7759/cureus.86011

**Published:** 2025-06-14

**Authors:** Fatima S Almohannadi, Rami A Mesk, Omar M Braizat, Mohamed B Ahmed, Abeer Alsherawi

**Affiliations:** 1 Department of Plastic and Reconstructive Surgery, Hamad Medical Corporation, Doha, QAT; 2 College of Medicine, QU Health, Qatar University, Doha, QAT

**Keywords:** ki-67, peripheral nerve sheath tumor, plexiform epithelioid schwannoma, s-100 protein, schwannomatosis, smarcb1/ini1

## Abstract

Plexiform epithelioid schwannoma (PES) is a rare, benign nerve sheath tumor that can resemble malignant tumors, making diagnosis challenging. We present the case of a 46-year-old Filipino man with a slow-growing mass on his chin. After surgical excision, histopathology confirmed it as PES. Immunohistochemistry supported the diagnosis, showing strong S-100 and SOX10 positivity. The patient recovered well, with no signs of local recurrence. This case highlights the importance of thorough pathological evaluation when diagnosing rare tumors like PES.

## Introduction

Schwannomas are benign tumors arising from neural crest cells, specifically originating within the peripheral nerve sheath. Epithelioid schwannoma is a rare histologic variant that poses diagnostic challenges due to its potential to mimic malignant neoplasms [1-4]. Plexiform schwannomas are another uncommon subtype, occasionally associated with neurofibromatosis and schwannomatosis, though they can also occur sporadically. Clinically, most schwannomas are asymptomatic and typically located within the dermis or subcutaneous tissue. These lesions are generally small, averaging around 1.5 cm in size, but can occasionally reach up to 6.8 cm [5]. Plexiform epithelioid schwannoma (PES) is an exceptionally rare and distinct variant of schwannoma, characterized by a plexiform growth pattern composed of epithelioid cells. Histological examination and immunohistochemical staining - particularly for markers such as S-100 protein and Ki-67 - are crucial for establishing a definitive diagnosis and excluding malignant potential. According to the literature, only one case has been reported to date exhibiting both plexiform and epithelioid features within a single schwannoma [6].

We present a rare case of PES manifesting as a lesion on the chin of a 46-year-old patient. Following surgical excision and immunohistochemical analysis, a definitive diagnosis of PES was made. This case is notable for its unusual histological features and adds to the limited body of literature on this rare entity.

## Case presentation

A 46-year-old Filipino male, previously healthy, working as a laborer and residing in Doha, Qatar, was referred by his primary care physician to the plastic surgery outpatient clinic in July 2024. He presented with a subcutaneous lesion located beneath the lower lip. The lesion measured approximately 1 × 1 cm, was non-tender, immobile, and rubbery in consistency. There were no overlying skin changes indicative of an inflammatory process. The patient reported that the lesion had been present for approximately one year, with a recent increase in size. Intraoral examination revealed no extension of the lesion into the oral cavity, and the overlying oral mucosa appeared intact. The initial clinical impression was that of a benign subcutaneous lesion; therefore, no imaging or biopsy was performed preoperatively.

**Figure 1:**
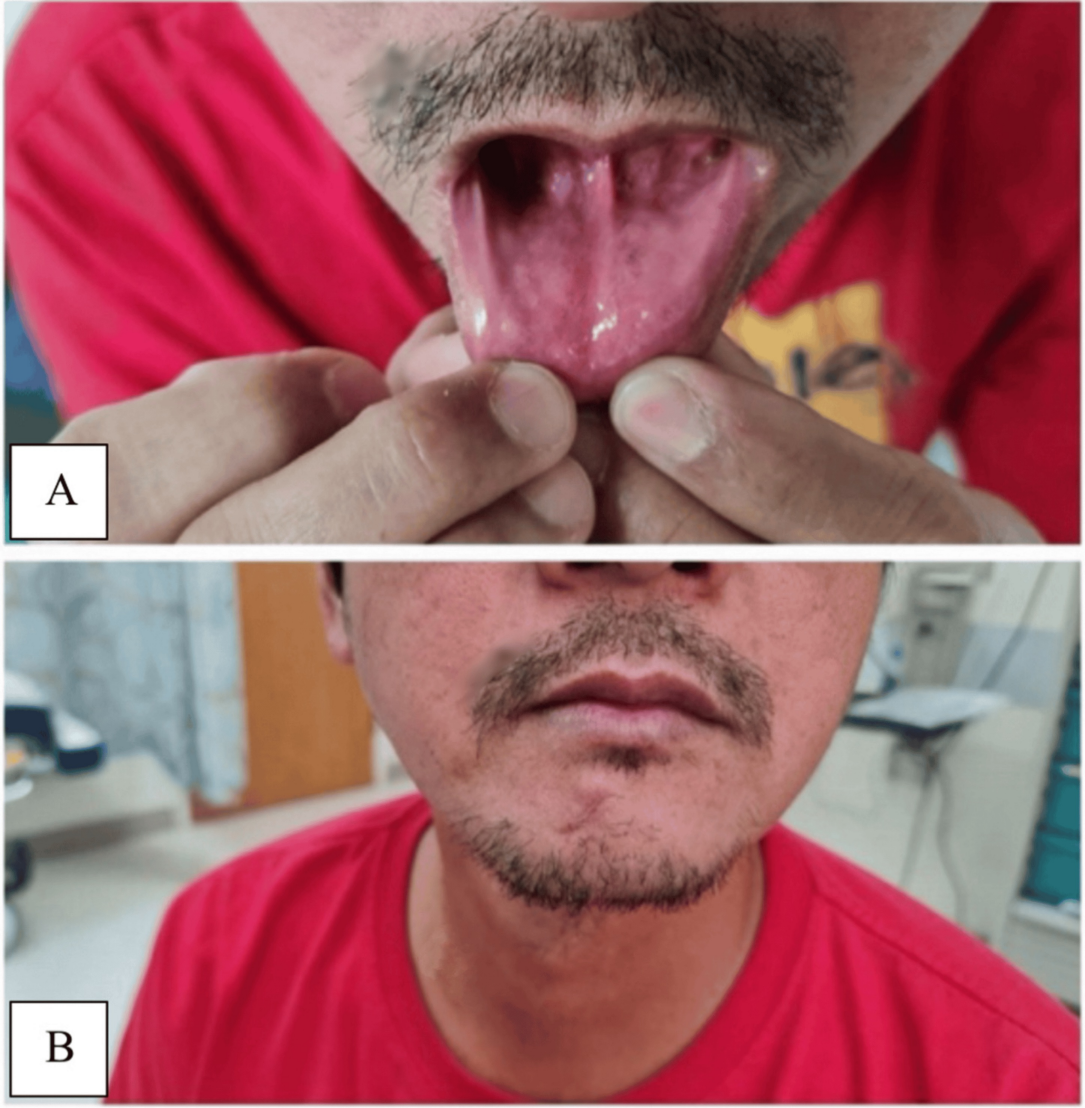
Three weeks postoperatively, with no evidence of intraoral extension (A). Surgical site at three weeks postoperatively showing well-healed scar (B).

**Figure 2:**
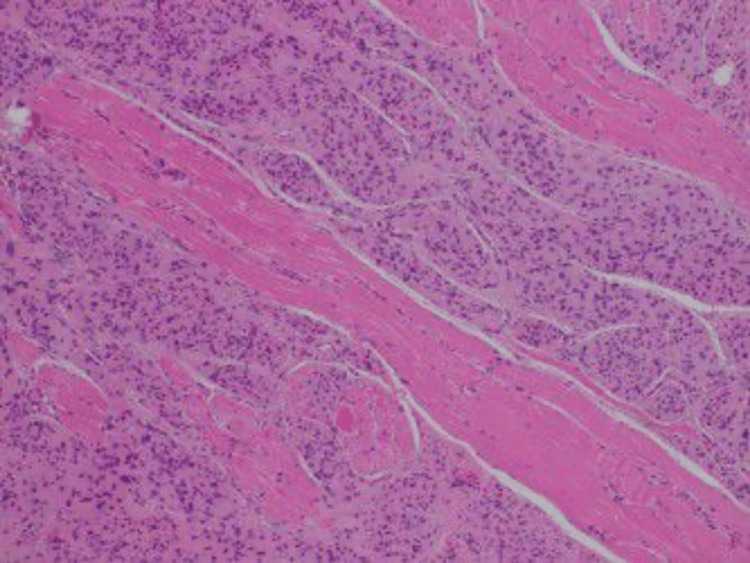
A deep dermal tumor composed of two populations of cells; spindle cells but mainly epithelioid cells surrounded by a thin capsule. There is no mitosis or atypia (H&E, 100×).

The patient was scheduled for a daycare procedure, during which the lesion was excised en bloc under local anesthesia. Primary closure of the wound was achieved. The postoperative course was uneventful, with no reported complications. Follow-up at three weeks demonstrated good healing of both the skin and oral mucosa, as shown in Figure 1. The excised specimen was initially sent for histopathological analysis to the local laboratory at Hamad Medical Corporation, Doha, Qatar. The microscopic examination of the specimen showed a tumor, which was intimately admixed with and seen to be growing in between skeletal muscle. Adipocytes and nerve fibers were also noted. The tumor cells were arranged in nests, nodules, and sheets. At certain foci, a whorling pattern was present too. The lesional cells were mostly oval to round in shape, although their cytoplasmic outlines were indistinct. The cytoplasm was eosinophilic and distinctly granular to wispy fibrillary in texture. The nuclei were mostly round to oval, but some were plump to slender and spindly. They showed a fair degree of anisonucleosis and nuclear margin irregularities. Their chromatin showed a variable texture. However, frank anaplasia or mitotic activity was absent. Some of the nuclei showed distinct intranuclear pseudoinclusions (Figure 2).

**Figure 3:**
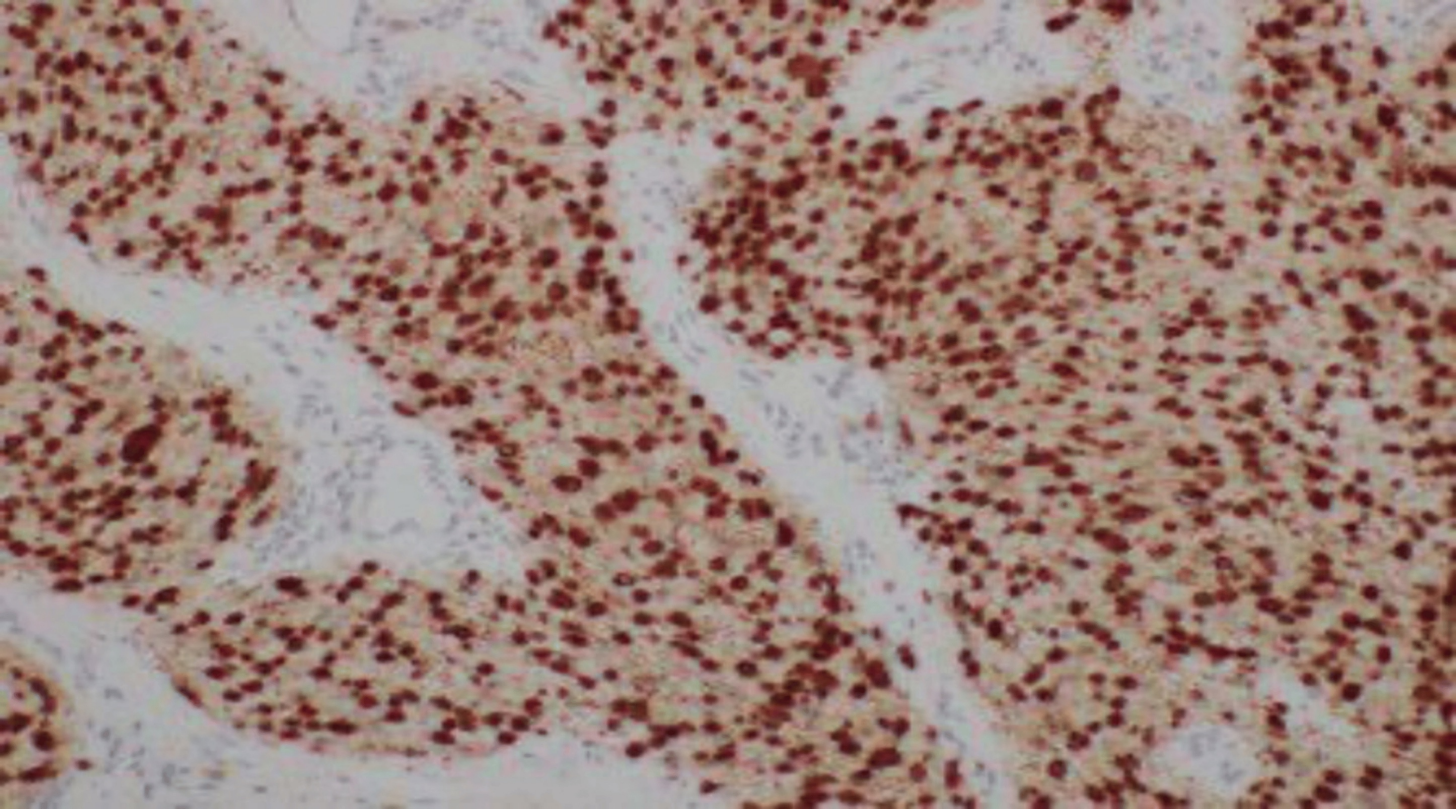
S-100 stain showing strong positivity (immunohistochemistry, 200×).

Regarding immunohistochemistry, tumor cells were S-100 and Sox-10 positive (Figure 3). INI-1 was preserved, and Ki-67 was <1%. The cells were negative for cluster of differentiation (CD)68, CD123, inhibin, CK-AE1/3, epithelial membrane antigen (EMA) (highlighted the perineural cells of trapped nerves), CD31, CD34, smooth muscle actin (SMA), synaptophysin, neural cell adhesion molecule (CD56), glucose transporter type 1 (GLUT-1), GATA-3, glial fibrillary acidic protein (GFAP), progesterone receptor, and melanosome. There was a focal equivocal positivity for neuron-specific enolase (NSE).

Given the rarity of the lesion, the specimen was subsequently referred to the Mayo Clinic’s Department of Histopathology for expert review and confirmation. The institution reported that the histologic sections revealed a tumor with a plexiform architecture, composed of epithelioid cells with nuclear atypia of the degenerative type, frequent nuclear pseudoinclusions, syncytial-appearing cytoplasm, and lacking conspicuous mitotic activity and necrosis. By immunohistochemistry, the lesional cells were diffusely and strongly positive for Schwann cell markers.

Interestingly, the INI-1 immunostain performed at Hamad hospital laboratory showed a focal mosaic pattern of expression in the tumor cells, a feature that has been reported in schwannomas associated with schwannomatosis and NF2. Therefore, clinical correlation to rule out a syndromic association was recommended. Based on histopathology and immunohistochemistry, a final diagnosis of peripheral nerve sheath tumor most compatible with an unusual PES was made.

## Discussion

PES is a rare histological variant of benign schwannomas arising from peripheral nerves. Unlike the conventional solitary, well-circumscribed schwannoma that typically features a spindle-cell morphology, this variant displays a plexiform growth pattern and epithelioid cell differentiation, which introduces significant diagnostic challenges [7]. These tumors are generally encapsulated, slow-growing, and asymptomatic in the early stages, making clinical detection difficult. Moreover, their histopathological features may mimic malignant neoplasms, further complicating diagnosis. Epithelioid schwannomas are infrequent and can arise at any skin site, usually presenting as solitary tumors [8]. They have clinical and histological resemblance to other neoplastic like epithelioid cell histiocytoma, epithelioid angiosarcoma, epithelioid leiomyosarcoma, and epithelioid malignant peripheral nerve sheath tumor. Therefore, biopsy remains essential for accurate diagnosis [9]. Histologically, these tumors demonstrate a plexiform arrangement of Schwann cells, which may be misleading during microscopic evaluation. Immunohistochemistry is critical, with strong S-100 positivity serving as a key diagnostic marker [10]. Surgical excision is the standard treatment and typically results in a low recurrence rate. To date, only one case has been reported in the literature with features similar to ours. Gao et al. described a 47-year-old female presenting with a solitary cutaneous lesion on the breast [6]. In both cases, the lesions were primary, occurred in adult patients, and posed diagnostic challenges due to their rarity and resemblance to malignancy. Gao et al. highlighted the complexity of diagnosing the plexiform variant of epithelioid schwannoma, which often requires expert histopathological review [11]. In our case, the specimen was referred to the Mayo Clinic for specialized histopathological assessment, reinforcing the value of expert consultation in diagnosing such rare entities. Similar to Gao’s case, our tumor exhibited a plexiform pattern and strong S-100 positivity. The tumor architecture in both cases was consistent with schwannoma, although the plexiform and epithelioid features complicated initial diagnosis. Interestingly, emerging evidence has highlighted the role of SMARCB1/INI1 in schwannoma pathogenesis. Approximately 40% of cases show mosaic loss of nuclear SMARCB1/INI1 expression [12], a pattern often associated with schwannomatosis and familial tumor syndromes. In contrast, sporadic solitary schwannomas tend to display more uniform INI1 expression. Ki-67 staining patterns also differ between familial and sporadic cases, with sporadic tumors often showing more intense staining [12]. Awareness of these immunohistochemical variations is important for distinguishing between syndromic and non-syndromic schwannomas. Both our case and that of Gao et al. were successfully managed by complete surgical excision, with no recurrence noted, supporting the generally favorable prognosis of PESs when adequately treated.

## Conclusions

PES represents an exceedingly rare variant of benign peripheral nerve sheath tumors, with distinctive histopathological and immunohistochemical features that may mimic malignancy. Accurate diagnosis depends on expert pathological evaluation, including immunoprofiling with markers such as S-100 and SOX10. Our case underscores the importance of maintaining a high index of suspicion for rare tumor variants in subcutaneous lesions and reinforces the critical role of expert consultation in establishing a definitive diagnosis. Complete surgical excision remains the treatment of choice and is associated with excellent outcomes and minimal risk of recurrence. By contributing to the limited literature on PES, this report highlights key diagnostic considerations and adds valuable clinical insight into its management.
